# Tübingen model study: large-scale introduction of rapid antigen testing in the population and the viral dynamics of SARS-CoV-2

**DOI:** 10.3389/fpubh.2023.1159622

**Published:** 2023-10-24

**Authors:** Jule Ayran, Carsten Köhler, Le Thi Kieu Linh, Gisela Schneider, Srinivas Reddy Pallerla, Florian Battke, Lisa Federle, Peter Martus, Peter G. Kremsner, Thirumalaisamy P. Velavan

**Affiliations:** ^1^Institute of Tropical Medicine, Travel Medicine and Human Parasitology, Competence Centre for Tropical Medicine Baden-Württemberg, Universitätsklinikum Tübingen, Tübingen, Germany; ^2^Vietnamese-German Center for Medical Research, VG-CARE, Hanoi, Vietnam; ^3^DIFAEM – German Institute for Medical Mission, Tübingen, Germany; ^4^Center for Genomics and Transcriptomics (CeGaT) GmbH, Tübingen, Germany; ^5^German Red Cross, Regional Centre Tübingen, Tübingen, Germany; ^6^Institute for Clinical Epidemiology and Applied Biometry, Universitätsklinikum Tübingen, Tübingen, Germany; ^7^Centre de Recherches Médicales de Lambaréné (CERMEL), Lambarene, Gabon; ^8^Faculty of Medicine, Duy Tan University, Da Nang, Vietnam

**Keywords:** rapid diagnostic tests, SARS-CoV-2, COVID-19, transmission, Tübingen model, B.1.1.7 lineage, variant of concern, epidemiology

## Abstract

Despite of contact restrictions, population mobility remains the main reason for the spread of SARS-CoV-2. The state of Baden-Württemberg (BW), Germany, approved a model study in Tübingen (TÜMOD) to evaluate how mandatory rapid diagnostic tests (RDT) could reduce transmission. Between 16 March and 24 April 2021, approximately 165,000 residents and visitors to the city were screened for SARS CoV-2 infection using Abbott Panbio™ COVID-19 Antigen rapid test device. We assessed incidences and recorded epidemiological characteristics in a subset of 4,118 participants recruited at three of the nine testing stations. PCR tests were performed in RDT-positives to determine the positive predictive value (PPV), and circulating variants of SARS-CoV-2 were identified by whole-genome sequencing. 2,282 RDT-negative samples were tested by pooled PCR to calculate the false negative rate (FNR). Viral load was compared between variants. 116 (3%) participants were positive by RDT, and of these, 57 (49%) were positive by PCR, 55 (47%) were negative. This resulted in a PPV of 51%. Of the 57 positives, 52 SARS-CoV-2 genomes were successfully sequenced. Of these, 50 belonged to the B.1.1.7 lineage, which had a high viral load (average Ct = 19). Of the 2,282 RDT negatives tested, all were PCR negative (FNR 0%). At the end of TÜMOD, the incidence in Tübingen, which was initially lower, had reached the incidence in the state of BW. While it is difficult to assess the impact of TÜMOD on incidence independent of confounding factors, further studies are needed to identify the effect of close-meshed testing on infection rates.

## 1. Introduction

Population mobility remains the main reason for the spread of SARS-CoV-2, and various forms of contact restrictions, including lockdowns, have been enacted in response to the COVID-19 pandemic, resulting in enormous economic burdens at both the individual and societal levels (1, 2). One way to limit this burden is to introduce a large-scale rapid SARS-CoV-2 test as a complementary measure to overcome the lockdown and enable a safe resumption of public life while containing the risk of an increase in infections.

SARS-CoV-2 rapid diagnostic tests (RDTs) have become an established infection control tool, with mass testing used as a control strategy and enabling population mobility. While PCR-based COVID-19 diagnosis is considered the gold standard for accurate diagnosis in hospitals, rapid SARS-CoV-2 tests (RDTs) have been preferred in communities. However, the sensitivity of RDTs in field studies was lower compared to PCR and largely depends on the time since infection or the presence of symptoms. In addition, their usefulness depends on the prevalence of SARS-CoV-2 infection in the population tested (3–5).

In Germany, several infection control measures were taken in early 2021, such as restricting retail, catering and cultural events, and limiting the size of public gatherings, while RDTs have been available since late 2020 (6). In this context, the state of Baden-Württemberg (BW) approved the implementation of a model study in the city of Tübingen (Tübingen Model Study, TÜMOD) (7). Its main objective was to assess how mandatory RDT testing could enable the reopening of public facilities without risking an increase in infections.

In addition to continuous monitoring of incidence, the study had three main objectives: (a) to conduct an epidemiological survey among individuals with positive and negative RDT results for SARS-CoV-2, (b) to compare SARS-CoV-2 RT-qPCR with RDT results and determine the positive predictive value (PPV) and false negative rate (FNR), (c) to conduct SARS-CoV-2 genome sequencing to determine viral lineages in circulation.

## 2. Context

During the TÜMOD study from 16 March to 24 April 2021 (7), retail stores, outdoor dining, body-related services, and cultural facilities could only be visited by clients holding a negative RDT certificate no more than 24 h old. To this end, nine testing stations throughout the city offered free RDT testing every day, performed by trained personnel. In case of a positive result, individuals were reported to the health authorities and subsequently referred for PCR testing. In case of a negative result, a “day ticket” was issued. Importantly, positive RDTs were not included in the 7-day incidence per 100,000 inhabitants, which is calculated from positive SARS-CoV-2 RT-qPCR only.

## 3. Details

### 3.1. Study cohorts for the evaluation of TÜMOD

**Figure 1:**
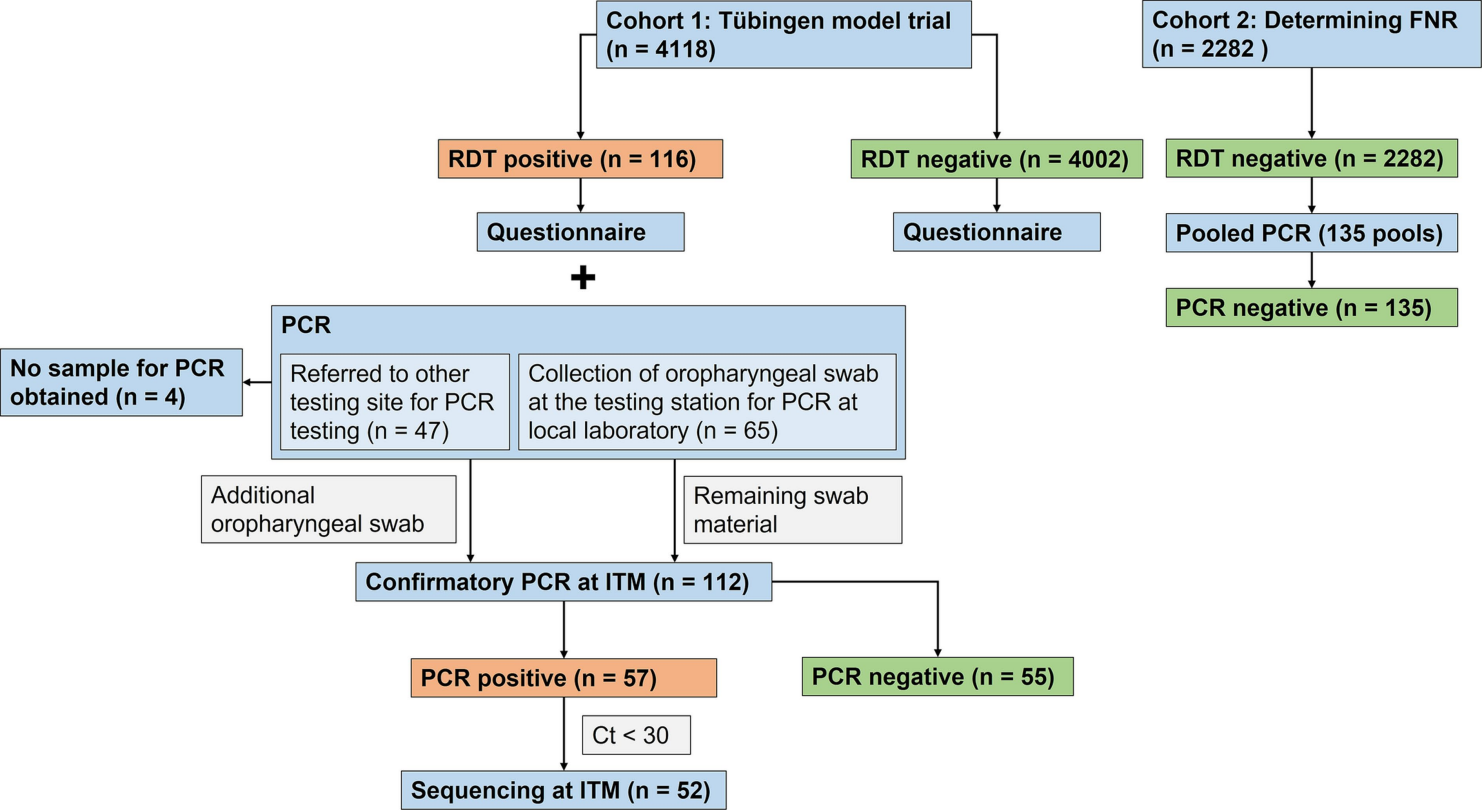
Flowchart illustrating the study design. During the Tübingen model study, participants undergoing RDT testing were included from 22 March to 19 May 2021 (Cohort1). After the study, cohort 2 was recruited at RDT testing sites for university staff members from 26 April to 22 June 2021, to determine the FNR (Cohort 2). FNR: False Negative Rate. RDT: Rapid Diagnostic Test; PCR: Polymerase Chain Reaction; ITM: Institute for Tropical Medicine, University of Tübingen; Ct: cycle threshold.

The purpose of the main study cohort (cohort 1) was to investigate epidemiological risk factors for infection, to determine the PPV and to determine the dominating lineages of SARS-CoV-2. 4,118 individuals with a negative or positive RDT result at a test station of TÜMOD were recruited (Figure 1). Individuals who were waiting in a queue for an RDT test at three test stations (“Market Square,”“Tourist Information Center,” and “Cultural Hall”) were asked to answer a pseudonymized questionnaire targeting epidemiological risk factors for infection. Interviews were conducted at the testing sites among RDT-negative (*n* = 4,002) and positive participants (*n* = 116). Those who tested RDT-positive at the site were subsequently required to undergo PCR testing at an official referral centre in the region. The samples and corresponding questionnaires were pseudonymized. Oro- or nasopharyngeal swabs from the RDT-positive individuals were sent to a local reference laboratory “Center for Genomics and Transcriptomics” (CeGaT) in Tübingen or to another laboratory in the region for a confirmatory RT-qPCR test. The Institute for Tropical Medicine of the University of Tübingen (ITM) was responsible for the independent confirmation of the PCR test. Similarly, it was responsible for sequencing the entire SARS-CoV-2 genome in all PCR-positive isolates. The institute for clinical epidemiology was responsible for the construction of questionnaires, which captured the risk factors listed in Table 1, as well as data acquisition and storage. The original questionnaire can be found in the Supplementary material.

**Table 1:** Epidemiological characteristics of the study population.

Variable	Uninfected (*n* = 4,057)	Infected (*n* = 57)		*p* value
Age	42	40		0.58^a^
	(SD = 18, n = 4,056)	(SD = 20, n = 48)		
Minor (< 18 years)	72/4056 (2%)	3/48 (6%)		0.13^b^
Gender				0.01^c*^
Female	2545/4045 (63%)	20/46 (43%)		
Male	1507/4045 (37%)	26/46 (57%)		
Diverse	2/4045 (0%)	0/46 (0%)		
Residence		Model study	Mar 22- Apr 1	0.53^b**^
Tübingen (City)	1871/4024 (46%)	26/45 (58%)	4/8 (50%)	
Tübingen (District)	362/4024 (9%)	14/45 (31%)	1/8 (12%)	
Baden-Württemberg (State)	1473/4024 (37%)	3/45 (7%)	2/8 (25%)	
Germany	318/4024 (8%)	2/45 (4%)	1/8 (12%)	
Employment	2533/4048 (63%)	30/47 (64%)		0.98^c^
Full time	1627/2518 (65%)	12/17 (71%)		0.67^c^
Home office	855/1979 (43%)	2/27 (7%)		**0.0002** ^c^
Full time	337/852 (40%)	0/2 (0%)		0.52^b^
Persons in household	2.9 (2.5, *n* = 4,051)	4.0 (5.6, *n* = 43)		0.35^a^
Children in household (< 18 y)	854/4037 (21%)	16/43 (37%)		0.02^c^
Number of children in household	0.4 (0.8, n = 4,037)	0.7 (1.1, n = 43)		0.008^a^
Public transportation				0.70^a^
Daily	341/3081 (8%)	4/49 (8%)		
Several times a week	309/3081(8%)	4/49 (8%)		
Approx. once a week	251/3018 (6%)	3/49 (6%)		
Less than once a week	463/3018 (11%)	9/49 (18%)		
Never	1654/3018 (41%)	25/49 (51%)		
Sharing a car (non-household)				0.19^a^
Daily	66/3020 (2%)	5/46 (10%)		
Several times a week	201/3020 (5%)	0/46 (0%)		
Approx. once a week	335/3020 (8%)	5/46 (10%)		
Less than once a week	724/3020 (18%)	15/46 (31%)		
Never	1694/3020 (42%)	21/46 (43%)		
Reason for visit^†^				
Shopping	1693/4055 (42%)	7/48 (15%)		**0.0003** ^c^
Tourism	675/4055 (17%)	0/48 (0%)		0.004^c^
Gastronomy	1191/4055 (29%)	3/48 (6%)		**0.0008** ^c^
Private	1028/4055 (25%)	15/48 (31%)		0.44^c^
Other	443/4055 (11%)	22/48 (46%)		**<0.0001** ^c^
COVID-19 vaccinated (at least once)	711/4052 (18%)	4/43 (9%)		0.15^c^
History of SARS-CoV-2 infection	178/4054 (4%)	2/43 (5%)		0.71^b^

Infected = RT-qPCR positive for SARS-CoV-2. uninfected = RDT negative or RDT positive and RT-qPCR negative for SARS-CoV-2. † Multiple answers were allowed. a = by Man-Whitney U test. b = by Fisher’s exact test. c = by Pearson’s chi squared test. *Excluding diverse. **Calculated for the comparable period Mar 22- Apr 1. Bonferroni-significant results (*p* < 0.002) are indicated in bold.

If PCR was performed at the local reference laboratory in Tübingen, the same swab material was used for RT-qPCR for independent confirmation at the ITM (*n* = 65). However, if PCR was analyzed at another laboratory outside of Tübingen, participants were asked to provide a second oropharyngeal swab at the testing site on the same day for analysis at the ITM (*n* = 47). Four RDT-positive individuals could not be confirmed by PCR at the ITM because no sample could be obtained. Participants with a positive RDT result were recruited from 22 March to 19 May 2021. Although the model study ended on 24 April, the testing facilities in Tübingen remained accessible to the Tübingen population and recruitment of RDT-positive participants continued until 19 May 2021.

As a high rate of false positive RDTs became apparent, it was decided to also investigate the false negative rate (FNR). Therefore, cohort 2 was recruited. Pooled PCR testing was performed in 2,282 RDT-negative individuals to determine the FNR. Since the model study had already ended and the demand at the test stations in the city had diminished, participants were recruited between April 26 and June 22, 2021 at five testing stations set up for University of Tübingen staff, where the same RDT was used. Oropharyngeal swabs were collected, and PCR testing was performed in pooled samples at CeGaT.

### 3.2. RT-qPCR and rapid diagnostic tests

Panbio™ COVID-19 Ag rapid test device (Abbott, Lake Country, IL, United States) was used. Collection of nasal swabs and the test were performed on site by trained personnel as by the manufacturer’s instructions. Results were available within 15–20 min. From RDT positive samples, RNA was extracted from oropharyngeal swabs using the QIAamp Viral RNA Mini Kit (Qiagen, Hilden, Germany). SARS-CoV-2 infection was confirmed by RealStar® SARS-CoV-2 real-time PCR targeting the S gene (Altona Diagnostics, Hamburg, Germany) using a LightCycler® 480 Instrument II (Roche Diagnostics, Mannheim, Germany) as described previously (8). RT-qPCR was performed within a median of two days (range: 0–11 days) after sample collection.

### 3.3. Sequencing and phylogenetic analysis

PCR-positive samples with cycle threshold (Ct) values <30 were subjected to whole genome sequencing on the Oxford Nanopore MinION™ (Oxford Nanopore Technologies, Oxford, UK) using 1,200 bp amplicon rapid sequencing (9). SARS-CoV-2 genomes were assembled using the ARTIC pipeline.^1^ All genomes were deposited in GISAID (10). Nextclade Web (version 1.7.4) identified nucleotide and amino acid substitutions in the sequences and the lineages were obtained using PANGOLIN (version 3.1.16, lineage version 2021-11-25) (11).

To reconstruct the phylogeny, we included sequences from this study (*n* = 52) as well as SARS-CoV-2 genomes from BW circulating during the study (*n* = 8), international sequences from the “nextstrain” dataset for Europe (*n* = 31), and the Wuhan Hu-1 reference sequence (NC_045512.2) retrieved from GISAID (10). Sequences were aligned using MAFFT (12). A maximum-likelihood phylogenetic tree was constructed in IQTREE (version 1.6.12) (13) under the GTR + F + R2 model using the BIONJ algorithm. 1,000 bootstrapping iterations were used. The tree was illustrated with the Interactive Tree of Life (iTOL) tool (version 6) (14). To assess whether the tree contains sufficient temporal information to allow analysis of development of mutations during the model study, root-to-tip regression analysis was conducted using TempEst (version 1.5.3) (15).

### 3.4. Data sources for epidemiology

The 7-day incidence per 100,000 inhabitants as well as the number of new infections with SARS-CoV-2 was obtained from the website of the Robert Koch Institute, the central federal authority for disease surveillance and prevention in Germany (16, 17). The population of BW and its districts in the second quarter of 2021 was retrieved from the State Statistical Office of BW (18).

### 3.5. Statistical analysis

Statistical analyses were conducted in R (version 4.0.0) (19). Population characteristics recorded in the questionnaires are reported as mean (Standard Deviation, SD) or proportion (percentage). Their distribution among infected and uninfected individuals as well as RDT false positives and true positives was compared by Man-Whitney U test, Pearson’s chi square test, or Fisher’s exact test. As this was an exploratory analysis, results reaching *p* = 0.05 were considered significant, while the Bonferroni-corrected threshold for significance would have been at 0.05/21 = 0.002 for infected vs. uninfected and 0.05/10 = 0.005 for true positives vs. false positives. For the observed PPV and FNR, two-sided exact 95% confidence intervals were calculated. The 7-day incidence per 100,000 inhabitants (rolling average) as well as the number of new infections per 100,000 inhabitants in a week were plotted and compared between Tübingen, the state of BW, and four other districts which were taken from the synthetic control model in the discussion paper by Diederichs et al. (20). These included the BW cities of Heidelberg (weight 0.431 in the synthetic control model) and Freiburg im Breisgau (weight 0.300) and the districts of Enzkreis (weight 0.254) and Heilbronn (weight 0.016). It was expected that incidence in Tübingen district would increase during TÜMOD because of increased testing, leading to the detection of more infections but not necessarily reflecting more infections in the district. To control for this, the number of true positive RDTs was estimated from the number of total positive RDTs based on a PPV of 0.5 for each week. Then, these true positive tests were subtracted from the number of new infections per 100,000 population in a week as they were possibly detected only because of the increased testing during the TÜMOD. The proportion of different SARS-CoV-2 lineages was compared by residence of infected individuals, and viral load by Ct values was compared between lineages in the Student’s t-test.

## 4. Results

### 4.1. Course of the model trial

The model study, which received nationwide media coverage, attracted many guests to Tübingen who wanted to participate in the social life which was not possible in their own towns. This led to overcrowding in the city and the city administration decided to limit day tickets for out-of-town guests to a maximum of 3,000 per day from 27 March. As this was not sufficient to limit overcrowding in popular localities, day tickets were limited to residents of the Tübingen district from 1 April (7). In response to an increase in incidence by then, the outdoor food service establishments had to be closed again on 6 April. Although incidences increased steadily in April, termination of the study was not deemed necessary by local authorities or the state government of BW. The study ended with the implementation of a new federal law for infection control, which was imposed on 23 April 2021, requiring contact restrictions and the closure of stores and food service establishments when the incidence (number of reported infections by PCR per 100,000 people in the past seven days) exceeded 100 (7).

### 4.2. Characteristics of the study population

The epidemiological characteristics of the study participants are shown in Table 1. Notably, the regulations as to who was allowed to participate in activities of the model study changed on 1 April 2021. Therefore, the place of residence can only be compared between uninfected and infected individuals until then. In this period, there was no major difference in the places of residence between the groups.

Two age peaks were observed, one around the mid-twenties and one around the 60s in both groups. Three of the 46 infected individuals were children (aged 7 to 16 years) belonging to two families. In these cases, the accompanying adult was also infected. In addition, the proportion of males among those infected was higher (*p* = 0.01). The infected more often lived with children in their household (*p* = 0.02) and had more children than the uninfected (*p* = 0.008). They also were less likely to work in home office (*p* = 0.0002). The proportion of people who had received at least one dose of SARS-CoV-2 vaccine was higher among the uninfected, although the difference was not significant (18% versus 9%, *p* = 0.15). There were no differences in the proportion of people with a positive test on a previous occasion between infected and uninfected people (*p* = 0.71).Remarkably, those infected were much more likely than those not infected to give other reasons for testing (none of the reasons “shopping,” “tourism,” “going to restaurants or private meetings” applied) (46% vs. 11%, *p* < 0.0001).

False and true RDT-positives were also compared (Table 2). False positives did not differ from the true positives in age (*p* = 0.15). The two groups did not differ in sex distribution (64% vs. 43%, *p* = 0.07). The reason “Other” was also much more prevalent among true positives (46% vs. 18%, *p* = 0.006), while “Shopping” was given less frequently (15% vs. 44%, *p* = 0.003). The number of participants who had received at least one dose of a vaccine against SARS-CoV-2 was high among false positives but was not significant (26% vs. 9%, *p* = 0.07).

**Table 2:** Epidemiological characteristics of true positives and false positives in RDT.

Variable	False positive (*n* = 55)	True positive (*n* = 57)	*p* value
Age	47 (SD = 19, *n* = 50)	40 (SD = 20, *n* = 48)	0.15^a^
Minor (< 18 years)	0/50 (0%)	3/48 (6%)	0.11^b^
Gender			0.07^c*^
Female	32/50 (64%)	20/46 (43%)	
Male	18/50 (36%)	26/46 (57%)	
Diverse	0/50 (0%)	0/46 (0%)	
Reason for visit^†^			
Shopping	22/50 (44%)	7/48 (15%)	**0.003** ^c^
Tourism	3/50 (6%)	0/48 (0%)	0.24^b^
Gastronomy	3/50 (6%)	3/48 (6%)	1^b^
Private	17/50 (34%)	15/48 (31%)	0.94^c^
Other	9/50 (18%)	22/48 (46%)	0.006^c^
COVID-19 vaccinated (at least once)	13/50 (26%)	4/43 (9%)	0.07^c^
History of SARS-CoV-2 infection	2/50 (4%)	2/43 (5%)	1^b^

False positive = RDT positive and RT-qPCR negative for SARS-CoV-2; True positive = RDT positive and RT-qPCR positive for SARS-CoV-2. ^†^Multiple answers were allowed. a = by Man-Whitney U test. b = by Fisher’s exact test. c = by Pearson’s chi squared test. *Excluding diverse. Bonferroni-significant results (*p* < 0.005) are indicated in bold.

### 4.3. Positive predictive value of the RDT

To assess the performance of the Abbott Panbio™ COVID-19 Ag rapid test under real-life conditions, swabs from participants with positive RDT were analysed by RT-qPCR. A PPV of 51% (57/112, 95% CI 41–60%) was observed (Supplementary Table). Among vaccinated individuals, the PPV was even lower at 24% (4/17, 95% CI 7–50%). As shown in Figure 1, a subset of 65 samples were subjected to RT-qPCR at CeGaT before sending the remaining swab materials to the ITM for confirmatory RT-qPCR and sequencing. While one sample, which was negative for SARS-CoV-2 infection at CeGaT, was not transferred to the ITM, all remaining 64 samples had concordant results in the RT-qPCR at both laboratories. One RDT positive sample had a Ct > 34 at both laboratories, which was considered a negative result for this analysis.

### 4.4. False negative rate of the RDT

A second cohort for the FNR determination was recruited at testing stations for members of the University of Tübingen only. A total of 2,282 individuals with a negative RDT result participated and were tested by RT-qPCR for SARS-CoV-2 in 135 Pools. None of the pools yielded a positive result, therefore the FNR in this cohort was zero (95% CI 0–0.0016). Notably, the incidence at the pool-testing sites was very low throughout the period, as none of the over 11,000 RDTs conducted there was positive. During TÜMOD, 0.15% of all RDTs performed at the testing sites in the city were positive. Assuming a similar prevalence at the pool-test stations, about 15 positive RDTs would have been expected there in total. Therefore, the prevalence at these sites must have been lower than at the testing stations in the city of Tübingen. It is therefore not possible to combine the two datasets to calculate sensitivity and specificity of the RDT.

### 4.5. SARS-CoV-2 incidence

**Figure 2:**
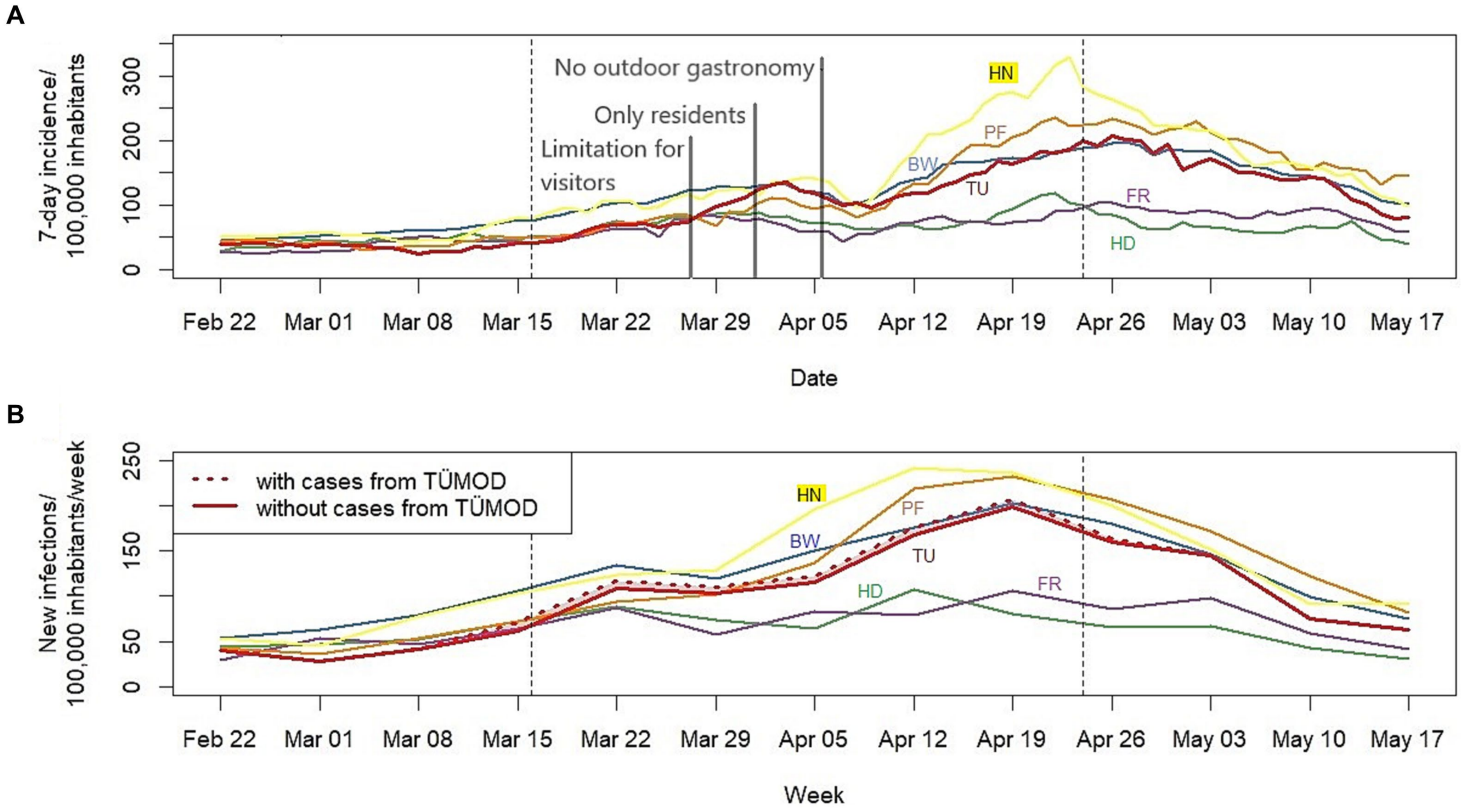
**(A)** Seven-day incidence and **(B)** new infections per week per 100,000 inhabitants for Tübingen, other districts, and Baden-Württemberg (BW). Dashed lines indicate beginning and end of the model study. Grey lines mark modifications of the regulations of the model trial as described in the main text. The number of new infections per 100,000 inhabitants per week without cases detected in the model study was calculated by subtracting the number of positive RDTs divided by 2, assuming a PPV of 0.5 throughout the model trail, from the new infections in Tübingen. For comparison, other districts of BW are included which were chosen for the synthetic control by Diederichs et al. (20): The city of Heidelberg (weight 0.431 in the synthetic control) and city of Freiburg im Breisgau (weight 0.300) as well as the district of Enzkreis (weight 0.254) and district Heilbronn (weight 0.016). TÜMOD = Tübingen model study. TU = district of Tübingen. HD = city of Heidelberg. FR = city of Freiburg im Breisgau. PF = district of Enzkreis. HN = district of Heilbronn. BW = Baden-Württemberg.

During the model study, a total of 230 positive RDTs was observed (0.15%, 114 of the 230 positives could not be reached for the study). Tübingen had one of the lowest incidences in BW before TÜMOD (41 per 100,000 over 7 days in Tübingen vs. 75 in BW on 16 March 2021, Figure 2). In the second half of March, incidences slowly rose in the district of Tübingen, peaking around 3 April (136/100,000 per week). Thereafter, there was a decrease in incidence both in Tübingen and in the other districts of BW, which may be related to a reporting delay due to the Easter holidays. Thereafter, incidences increased steadily, reaching a maximum of 207 in Tübingen on 26 April. A similar trend was observed for BW, Enzkreis, and Heilbronn, but not for the cities of Heidelberg and Freiburg im Breisgau. At the end of the model study, the 7-day incidence in Tübingen was similar to that of BW (199 vs. 188 on 24 April 2021). However, if one subtracts the new infections that were possibly only detected due to the model study, the number of new infections per 100,000 inhabitants per week in Tübingen never surpassed the one in BW (Figure 2). Still, the increase in infections was steeper in Tübingen than in the whole state of BW, leading to an assimilation of 7-day incidence in Tübingen to that of the state during the model study.

### 4.6. SARS-CoV-2 lineages

Of the 57 samples positive for SARS-CoV-2, 52 were successfully sequenced. Of these, 50 (96%) belonged B.1.1.7 lineage, while the other two samples belonged to the B.1.1.318 and B.1.258.17 lineages. Of 50 individuals infected with B.1.1.7 lineage, 22 individuals were from the city of Tübingen (56%), 13 from the district of Tübingen (33%), and two from BW and other parts of Germany (5% each). The individual carrying the B.1.1.318 lineage came from a neighbouring district in BW. The individual infected with B.1.258.17 was also from another district. Ct values were compared between the 52 samples from TÜMOD, and 13 SARS-CoV-2 positive samples collected in early 2020 in another study (21), which were classified as B.1 (12/13, 92%) and B.1.1.285 (1/13, 8%). The mean Ct value of B.1.1.7 samples was 19, whereas it was 25 for the other lineages (Supplementary Figure 1). The difference between these two groups was significant (*p* < 0.01).

**Figure 3:**
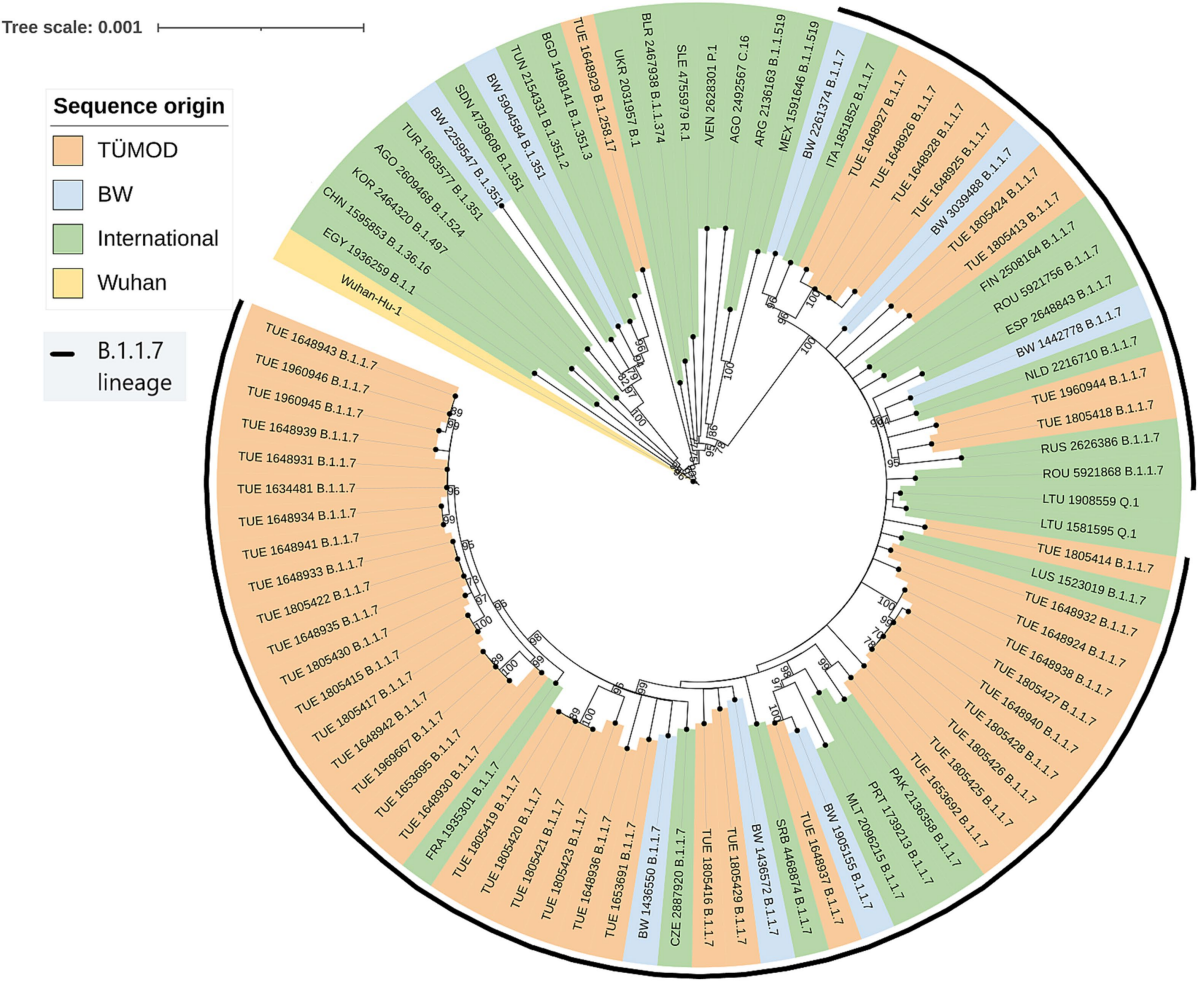
Maximum-likelihood phylogenetic tree. The tree was constructed under the GTR + F + R2 model using the BIONJ algorithm with 1,000 bootstrapping iterations. Bootstrapping values >70 are printed on the nodes. TÜMOD = Tübingen model project (*n* = 52). BW = Baden-Württemberg (*n* = 8). International = “nextregions” dataset (*n* = 31). Wuhan = Wuhan-Hu-1 reference sequence (NC_045512.2). GISAID accession numbers can be found in the Supplementary material.

Reconstruction of phylogeny revealed a cluster of sequences classified as B.1.1.7 and Q.1, in which samples from Tübingen and BW formed a subgroup, being evolutionary closer to each other than to samples from the international context sequences (Figure 3). Root-to-tip regression showed a correlation of time and evolutionary distance (R2 = 0.32), but this was likely caused by the reference sequence, which has been dated much earlier. All other sequences form a cluster without recognizable slope around the regression line between March and May 2021 (Supplementary Figure 2). Therefore, the phylogenetic tree likely did not contain sufficient temporal information to conduct a meaningful analysis on phylodynamics during TÜMOD.

## 5. Discussion

The study aimed to evaluate how mandatory large scale rapid diagnostic testing can act as a complementary measure to overcome the lockdown and enable a safe resumption of public life while containing the risk of an increase in infections. The survey conducted among infected and uninfected individuals showed that infected individuals more frequently were male, lived in larger households, lived with children, and did not work in home office. The role of children in the transmission of SARS-CoV-2, who rarely develop severe disease but could act as asymptomatic carriers, has been extensively discussed (22, 23). However, the infected persons often made use of the testing opportunities because they already had a reason to believe that they were infected. This might explain the fact that they more often stated “other reasons for testing.” Informal communication between subjects and examiners revealed that they often had contact with an infected person or had symptoms of SARS-CoV-2 infection. Since persons already suspected of being infected should be separated from tourists and shoppers at the testing sites to reduce the risk of transmission while waiting for the test, it is important to set up separate testing sites for them. This was done at the referral PCR testing centre, but apparently not communicated clearly enough to the community.

When comparing false positive and true positive RDT results, it was found that people who had received at least one dose of the SARS-CoV-2 vaccine were strongly represented among the false positive results. Detection of antigens derived from a very recent vaccination is highly unlikely (24) and as the Abbott Panbio™ COVID-19 Ag rapid test also targets the N protein of SARS-CoV-2, cross-reaction with vaccines containing the S protein or its mRNA can be ruled out. Up to now, we did not find an explanation for this observation. It might be due to the fact that the vaccinated were less likely to be infected, whatever the RDT result might have been. The PPV was calculated as a measure of RDT accuracy. A PPV of 51% in this study reveals that half of all RDT-positive cases have been reported to the health authorities and were quarantined unnecessarily. Among vaccinated individuals, the PPV was even lower, where three quarters of the RDTs were false positive. Other evaluation studies by Wagenhäuser and colleagues compared RDTs with RT-qPCR in 5,068 screening tests in a hospital setting and reported a PPV of 97% in patients with COVID-19 symptoms and of 29% in patients without or with atypical symptoms (4). Another study found a PPV >90% in symptomatic patients or their asymptomatic contacts (3). Although measures of specificity and sensitivity vary widely between studies (25), a low PPV means a low prevalence. This is consistent with our finding that participants who reported a reason for testing other than shopping, tourism, visiting restaurants or private gatherings had more true positives, which may indicate a higher prevalence in this subgroup.

There are various possible reasons for false positive results in RDTs, e.g., incorrect test performance, cross-contamination, cross-reaction with other antigens or interfering substances leading to low specificity (26). However, performance evaluation studies have shown high specificity under real-life conditions (25). As the process of sample collection and testing was streamlined and the sample was collected by one person and then passed on to another who performed the test, improper use cannot be ruled out but is unlikely to fully explain the low PPV. Furthermore, in a second cohort, no false negative results were detected among 2,282 RDT-negative individuals. Although this could be a sign of a very high sensitivity of the test, which could not be demonstrated in field studies (25), the prevalence of SARS-CoV-2 might have been very low in this cohort, represented exclusively by university staff - a selected group that might have been more privileged in terms of exposure to the virus than the average visitor to the Tübingen model project. The vaccination rate could also be higher in this subgroup.

The alpha variant of concern (line B.1.1.7) was identified in most SARS-CoV-2 isolates from TÜMOD participants. Higher viral loads (27) and infectivity (28) were observed in infections with this variant, which replaced the B.1 lineage in Europe in early 2021. The number of SARS-CoV-2 infections in the districts of Tübingen increased fivefold during the study period and reached an equilibrium as in the federal state of BW in April. As this trend was observed in most areas of Germany between March and April 2021, it is difficult to draw conclusions about a causal relationship with the model project.

The incidences of SARS-CoV-2 infection in the district of Tübingen was analysed and compared to a synthetic control model by Diederichs and colleagues (20). By creating a “virtual twin” of the Tübingen district, where no model study was implemented, they aimed to determine the effect which TÜMOD had on the incidences, independent of other factors. The authors compared the 7-day incidence per 100,000 inhabitants between Tübingen and their synthetic control and calculated the incidence in Tübingen without cases detected only due to the model study, assuming a PPV of 0.5, an estimate, which was confirmed by the present study. They found that there were fewer cases in Tübingen at the beginning of the model study than would have been expected without the study, but that the case numbers rose in early April more than can be explained by increased testing alone. Therefore, it can be argued that TÜMOD was responsible for a modest increase in documented infections in early April.

It is noteworthy that this analysis contains data of the entire district of Tübingen, not only of the city itself. As highlighted in the final report of the model project submitted to the state of BW, the incidence in the city of Tübingen, where 40% of the district’s inhabitants live, was consistently lower than in the district of Tübingen as a whole (7). Since the day ticket was mainly used by residents of the city, the increase in infections in the district can only be partially related to the model project. In this context, it is remarkable that the incidences in the cities of Heidelberg and Freiburg hardly increased in April and had a much lower peak value than in BW or the districts of Enzkreis and Heilbronn (Figure 2). Heidelberg and Freiburg are small cities in BW with universities and a rather young population, similar to Tübingen, also reflected by larger weights in the synthetic control model by Diederichs et al. (20). Therefore, the city of Tübingen might have performed quite differently than the remaining district of Tübingen, showing infection dynamics closer to Heidelberg and Freiburg. In addition, other unique local events complicate the comparison of infection dynamics between different areas and populations. The peak around 1 April in Tübingen district can be attributed to an outbreak in a reception facility for refugees and is therefore most likely not related to the model study (7).

Limitations included the fact that only a fraction of RDT positives could be interviewed, so that their group was significantly smaller than that of RDT negatives. In addition, the negative ones were only interviewed during a short period at the beginning of the study. Further, the two study cohorts, the first cohort for PPV determination and epidemiological analysis and the second cohort for FNR analysis, were not recruited from the same parent population due to organizational restrains. Therefore, they cannot easily be compared to each other and sensitivity and specificity of the RDT cannot be calculated. Furthermore, no epidemiological data at the level of the city of Tübingen were available for a detailed evaluation of the infection incidence.

## 6. Conclusion

The Tübingen model project intended to assess the effects of cautious revival of social and economic life under close-meshed testing. During the model project, incidence increased to the level of the state of BW. It is difficult, however, to draw firm conclusions about the impact of TÜMOD on district incidence due to several possible confounders and the natural lack of a “control group,” which would have to consist of a second, comparable city without a model trial. Still, the model trial allowed the city to return to a freer public life to some extent. The implementation of broad-based testing strategies in low-prevalence settings also resulted in a low PPV, which must be accounted for in the assessment of the endemic situation. In addition, informing the population about the intended use of the test offers is crucial to direct people with suspected infection to separate test centres. Epidemiological surveys suggested an increased risk of infection for males and people living with children in their household, and a protective role of working in home office. The dominating lineage was B.1.1.7 during the whole model project.

The vaccination rate has now reached 75% or more, but new variants such as Omicron and its sub lineages are dominating the community. These new factors could have a major impact on the sensitivity and PPV of RDTs and must equally be given consideration in future testing strategies. In summary, this is an interesting study model that the city of Tübingen has carried out with the perspective of keeping business and social life alive in the city while accounting for the risk of an increase in infections.

## Data availability statement

The datasets presented in this study can be found in online repositories. The names of the repository/repositories and accession number(s) can be found in the article/Supplementary material.

## Ethics statement

The studies involving humans were approved by Ethics Commission of the Medical Faculty of the Eberhard-Karls University and the University Hospital of Tübingen. The studies were conducted in accordance with the local legislation and institutional requirements. Written informed consent for participation in this study was provided by the participants' legal guardians/next of kin.

## Author contributions

JA and SP: investigation, formal analysis, and writing – original draft. CK, PM, PK, and LF: conceptualization, methodology, and writing – review & editing. LL and GS: investigation. FB: investigation and writing – review & editing. TV: conceptualization, methodology, formal analysis, and writing – original draft. All authors contributed to the article and approved the submitted version.

## Footnotes

^1^ https://github.com/artic-network/fieldbioinformatics
