# Supplementary material for: Tübingen model study: large-scale introduction of rapid antigen testing in the population and the viral dynamics of SARS-CoV-2
Source: Front Public Health. 2023 Oct 24;11:1159622. doi: 10.3389/fpubh.2023.1159622 (PMC10628735; doi:10.3389/fpubh.2023.1159622)
Supplement: Supplementary file 1 [file Table_1.docx]

**Supplementary Table:** RDT and RT-qPCR results during the model study.

| **All**  RDT positive  RT-qPCR positive  RT-qPCR negative  RT-qPCR result unknown  PPV | n = 116  n = 57  n = 55  n = 4  51% (41 – 60%) |
| --- | --- |
| **Vaccinated**  RDT positive  RT-qPCR positive  RT-qPCR negative  RT-qPCR result unknown  PPV | n = 17  n = 4  n = 13  n = 0  24% (7 – 50%) |
| **Unvaccinated**  RDT positive  RT-qPCR positive  RT-qPCR negative  RT-qPCR result unknown  PPV | n = 84  n = 43  n = 37  n = 4  54% (42 – 65%) |

Two-sided exact 95% confidence intervals are given in brackets. RDT: rapid diagnostic test. RT-qPCR: reverse transcriptase quantitative PCR. PPV: positive predictive value
