# Supplementary material for: Tübingen model study: large-scale introduction of rapid antigen testing in the population and the viral dynamics of SARS-CoV-2
Source: Front Public Health. 2023 Oct 24;11:1159622. doi: 10.3389/fpubh.2023.1159622 (PMC10628735; doi:10.3389/fpubh.2023.1159622)
Supplement: Supplementary file 3 [file Image_1.pdf]

Supplementary Figure 1: **Violin plot of cycle threshold (Ct) values in RT-qPCR**.

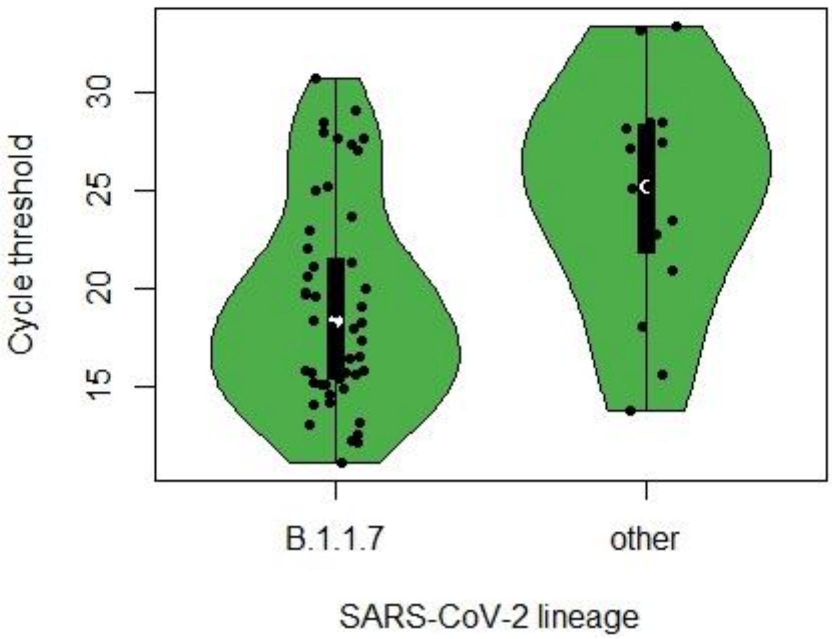

Ct values for lineage B.1.1.7 (n = 50) and other lineages. Ct = cycle threshold. Other lineages contain B.1 (n = 12), B.1.1.258 (n = 1), B.1.1.318 (n = 1), and B.1.258.17 (n = 1).
