# Supplementary material for: Tübingen model study: large-scale introduction of rapid antigen testing in the population and the viral dynamics of SARS-CoV-2
Source: Front Public Health. 2023 Oct 24;11:1159622. doi: 10.3389/fpubh.2023.1159622 (PMC10628735; doi:10.3389/fpubh.2023.1159622)
Supplement: Supplementary file 4 [file Image_2.pdf]

Supplementary Figure 2: Root-to-tip regression.

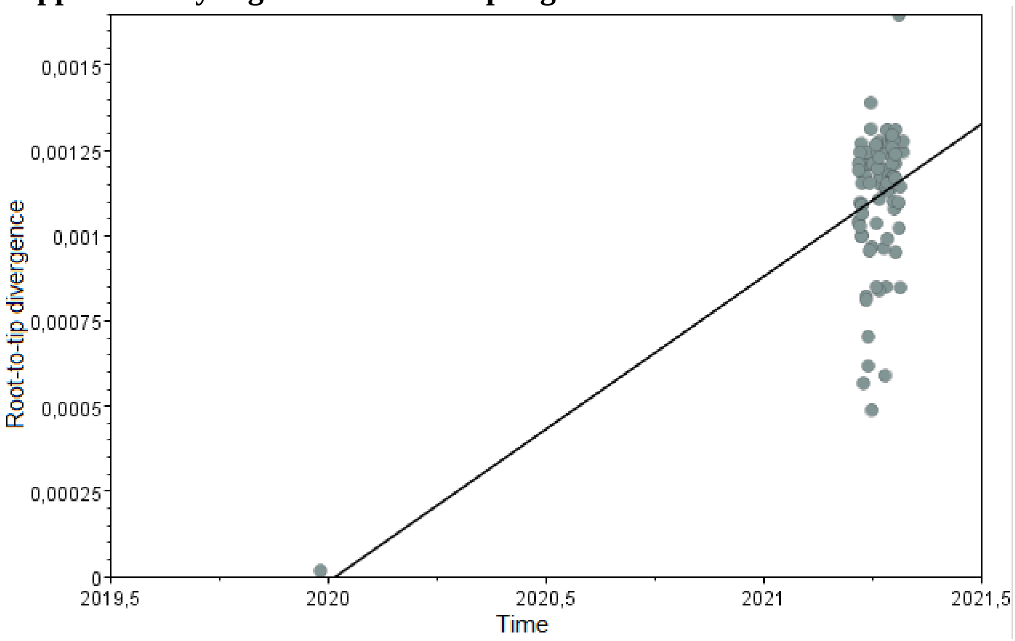

Root-to-tip regression analysis was conducted on the maximum-likelihood phylogeny shown in Figure 4. Slope = $8.96x10^{-4}$. R squared = 0.32. Residual mean squared = $3.48x10^{-8}$. As all SARS-CoV-2 sequences expect the Wuhan-Hu-1 form a cluster with no recognizable slope around the regression line, the phylogeny does not to contain significant information temporal development of the virus.
