# Supplementary material for: Tübingen model study: large-scale introduction of rapid antigen testing in the population and the viral dynamics of SARS-CoV-2
Source: Front Public Health. 2023 Oct 24;11:1159622. doi: 10.3389/fpubh.2023.1159622 (PMC10628735; doi:10.3389/fpubh.2023.1159622)
Supplement: Supplementary file 5 [file Image_3.pdf]

Wissenschaftliche Begleituntersuchung: Universitätsklinikum Tübingen

**Ort:**	
**Datum:**	

		Kategorien
**ID (wird vom Gesundheitsamt ausgefüllt)**		
**Geburtsjahr:**		*JJJJ*
**Geschlecht:**		*w / m / d*
**PLZ:**		
**Anzahl Personen im Haushalt:**		
**Anzahl Kinder (< 18J) im Haushalt:**		
**Berufstätig:**		*ja / nein*
		bei "ja": Voll-/Teilzeit in %
**Beruf:**		*Freitext*
**Homeoffice:**		*ja / nein*
**SchülerIn/StudentIn:**		*ja / nein*
**Wie oft nutzen sie öffentl. Verkehrsmittel?**		*1 = täglich*<br>*2 = mehrmals pro Woche*<br>*3 = ca 1x pro Woche*<br>*4 = wenigers als 1x pro Woche*<br>*5 = nie*
**Wie oft fahren Sie Auto mit mehreren Personen**<br>(Familienmitgleider zählen nicht)		*1 = täglich*<br>*2 = mehrmals pro Woche*<br>*3 = ca 1x pro Woche*<br>*4 = wenigers als 1x pro Woche*<br>*5 = nie*
**Haben Sie einen Schnelltest in Tübingen erhalten?**		*ja / nein*
**Wenn ja, Datum des Schnelltests**		*TT/MM/JJ*
**Wenn ja, Ergebnis des Schnelltests**		*positiv/negativ*
**Grund des Besuchs:**		
Nur ausfüllen, wenn Sie TÜ im Rahmen des Modellversuchs "Öffnen mit Sicherheit" besucht haben **Shopping**		*ja / nein*
**Tourismus**		*ja / nein*
**Gastronomie**		*ja / nein*
**Privat**		*ja / nein*
**Sonstiges**		*ja / nein*
**Bereits geimpft:**		*1 mal/2 mal/nein*
**Schon einmal postiv getestet, egal wo:**		*ja / nein*

Bitte senden an:

Prof. Dr. Peter Martus
Institut für Klinische Epidemiologie, UKT Tübingen
Silcherstraße 5
72076 Tübingen
